# A Predictive Assessment of Ochratoxin A’s Effects on Oxidative Stress Parameters and the Fermentation Ability of Yeasts Using Neural Networks

**DOI:** 10.3390/foods13030408

**Published:** 2024-01-26

**Authors:** Željko Jakopović, Davor Valinger, Karla Hanousek Čiča, Jasna Mrvčić, Ana-Marija Domijan, Iva Čanak, Deni Kostelac, Jadranka Frece, Ksenija Markov

**Affiliations:** 1 Laboratory for General Microbiology and Food Microbiology, Faculty of Food Technology and Biotechnology, University of Zagreb, Pierottijeva 6, 10000 Zagreb, Croatia; zjakopovic@pbf.hr (Ž.J.); icanak@pbf.hr (I.Č.); dkostelac@pbf.hr (D.K.); kmarko@pbf.hr (K.M.); 2 Laboratory for Measurement, Control and Automatisation, Faculty of Food Technology and Biotechnology, University of Zagreb, Pierottijeva 6, 10000 Zagreb, Croatia; dvalinger@pbf.hr; 3 Laboratory for Fermentation and Yeast Technology, Faculty of Food Technology and Biotechnology, University of Zagreb, Pierottijeva 6, 10000 Zagreb, Croatia; khanousekcica@pbf.hr (K.H.Č.); jmrvcic@pbf.hr (J.M.); 4 Department of Pharmaceutical Botany, Faculty of Pharmacy and Biochemistry, University of Zagreb, Schrottova 39, 10000 Zagreb, Croatia; adomijan@pharma.hr

**Keywords:** artificial neural networks, fermentation ability, oxidative stress, ochratoxin A, wine yeasts

## Abstract

The aim of this paper was to examine the effect of different OTA concentrations on the parameters of oxidative stress (glutathione (GSH) and malondialdehyde (MDA) concentrations) and glucose utilization in ethanol production by wine yeasts. In addition to the above, artificial neural networks (ANN) were used to predict the effects of different OTA concentrations on the fermentation ability of yeasts and oxidative stress parameters. The obtained results indicate a negative influence of OTA (4 µg mL^−1^) on ethanol production after 12 h. For example, *K. marxianus* produced 1.320 mg mL^−1^ of ethanol, while in the control sample 1.603 µg mL^−1^ of ethanol was detected. However, after 24 h, OTA had no negative effect on ethanol production, since it was higher (7.490 and 3.845 mg mL^−1^) in comparison to control samples. Even low concentrations of OTA affect GSH concentrations, with the highest being detected after 12 and 24 h (up to 16.54 µM), while MDA concentrations are affected by higher OTA concentrations, with the highest being detected at 24 h (1.19 µM). The obtained results with the use of ANNs showed their potential for quantification purposes based on experimental data, while the results of ANN prediction models have shown to be useful for predictions of what outcomes different concentrations of OTA that were not part of experiment will have on the fermentation capacity and oxidative stress parameters of yeasts.

## 1. Introduction

Mycotoxins are natural compounds whose chemical structures and biological activity differ from each other [1,2]. They are not necessary for the growth, development and reproduction of molds, but they help them in their defense against insects, other microorganisms, animals or competitive molds [3,4]. Since the initial isolation of OTA, subsequent research has revealed its presence in a diverse array of foodstuffs, encompassing cereals, spices, dried fruits, nuts and oil seeds, coffee, beer, grapes and wine [5]. Furthermore, OTA has been detected in meat products derived from animals that have been exposed to contaminated feed [6]. OTA stands out as one of the most potent representatives among the ochratoxin group of mycotoxins, exerting deleterious effects on multiple organ systems, therefore indicating a significant threat to both human and animal health [7]. The toxic effect has been proven especially in organs such as the kidneys, liver and circulatory system. Notably, it has been hypothesized to be a contributing factor to the incidence of Balkan endemic nephropathy, a severe and protracted kidney ailment with a potential link to the development of urinary tract tumors [5]. The categorization of OTA as a possible human carcinogen (Group 2B) by the IARC [8] underscores the imperative for the robust monitoring and regulation of OTA in food products to safeguard public health. The toxicological impact of OTA and its intermediates is contingent upon a multitude of variables, with paramount significance attributed to factors such as OTA concentration, duration of exposure, and the characteristics of the host organism [9]. Since yeasts are important microorganisms in biotechnological production (of ethanol for example) and in the production of fermented food and beverages, the presence of mycotoxins might affect their abilities and utilization [10]. Existing research has revealed a range of epigenetic mechanisms associated with OTA toxicity, principally encompassing processes related to oxidative stress, cellular proliferation, and perturbations in cell signaling and division dynamics [11,12,13,14]. Even though OTA can affect yeasts’ abilities and utilization, those same yeasts (or their components) can be used for the biodegradation of OTA-contaminated matrices. The biodegradation of OTA can be categorized into three types: (i) growth inhibition of fungi that synthesize OTA; (ii) biosorption of OTA by cells or their components; and (iii) enzymatic degradation of OTA into less toxic compounds [15]. Similarly, previous research has shown that OTA affects yeast cell count, which can be associated with the binding of OTA to the cell wall of yeasts, and cell diameter which could be related to the penetration of OTA into yeast cells [16]. Within cellular systems, a defensive apparatus operates to regulate the levels of free radicals, encompassing both enzymatic defenses such as superoxide dismutase (SOD), catalase (CAT), and glutathione peroxidase (GPx), as well as non-enzymatic molecules like glutathione (GSH) [17]. Notably, GSH, a cysteine-rich protein, assumes a pivotal role as a primary antioxidant within cellular contexts, conferring protection against oxidative stress and being regarded as one of the principal cellular antioxidants [18]. On the other hand, malondialdehyde (MDA) arises as a byproduct of lipid peroxidation resulting from reactions between free radicals and polyunsaturated fatty acids [19]. Both GSH and MDA can serve as indicative markers of oxidative stress within yeast cells. Experimental investigations have substantiated that in vitro exposure to OTA induces an increased production of free radicals, detectable through an increase in MDA concentration or a decrease in GSH levels [20]. In their research [10] have detected a higher level of MDA in samples exposed to a higher concentration of OTA (8.4 µg L^−1^), but no effect was detected on GPx and GST (Glutathione S-Transferase) in the yeast *Saccharomyces cerevisiae*. Furthermore, owing to its structural resemblance to phenylalanine, OTA hampers protein synthesis and may impede the function of enzymes utilizing phenylalanine as a substrate, thereby interfering with glucose metabolism [9]. Therefore, the aim of this research was to investigate effect of different concentrations of OTA (2 and 4 µg mL^−1^) on GSH and MDA concentrations as parameters of oxidative stress as well as glucose utilization during ethanol production by yeasts *S. bayanus*, *K. marxianus*, *H. uvarum* and *P. guilliermondii*. In recent years, more and more scientists have turned to artificial intelligence in order to get better predictive models. The main reason is the ability of artificial neural networks (ANN) to predict nonlinear models that sometimes occur during the cultivation of microorganisms [21]. ANNs can perform many parallel computations for data processing [22] and have therefore been applied to develop models that could estimate the growth parameters of bacteria or fungi [23,24] or the production of metabolites [25]. The application of ANNs to predict the effects of OTA concentrations on fermentation ability and oxidative stress parameters of yeasts may be useful and may refer to a better control of OTA in everyday commodities due to its ubiquity. Therefore, the objectives of this study encompassed the assessment of two distinct OTA concentrations on oxidative stress parameters, namely GSH and MDA levels, along with the quantification of residual glucose concentrations and the concurrent determination of ethanol production during the cultivation of select yeast strains.

## 2. Materials and Methods

### 2.1. Microorganisms

The following wine yeasts were selected to determine the effects of OTA on the fermentation ability and parameters of oxidative stress: *Saccharomyces bayanus* 8, *Kluyveromyces marxianus* DS12, *Hanseniaspora uvarum* S138 and *Pichia guilliermondii* ZIM 624, obtained from the Microorganism Collection of the Faculty of Food Technology and Biotechnology, University of Zagreb. Yeast cultures were stored at −20 °C in glycerol until the experiments were set up.

### 2.2. OTA Standard

The OTA standard utilized in this study was obtained in the form of crystalline material (Sigma-Aldrich, Merck, MO, USA). Stock solutions in concentrations of 2 mg mL^−1^ and 4 mg mL^−1^, were prepared by using 96% ethanol, which were stored until use at −20 °C. The working concentrations of OTA used in the experiments were prepared by introducing OTA into the yeast growth medium.

### 2.3. Yeast Cell and Sample Preparation

Selected yeast strains, which were stored at −20 °C in malt broth (Biolife, Milan, Italy) with 30% (*v*/*v*) glycerol, were revitalized by inoculation into fresh malt broth and incubated at 28 °C for 48 h. After incubation, the number of live yeast cells was determined, which was 10^7^–10^8^ cells mL^−1^.

One mL of suspension of each yeast strain was inoculated into 3 Erlenmeyer flasks with 100 mL of YPG (Yeast Peptone Glucose) broth (Biolife, Milan, Italy), to which OTA dissolved in ethanol was added to final concentrations of 2 µg mL^−1^ or 4 µg mL^−1^, and only ethanol was added to the control sample instead of OTA dissolved in ethanol. The samples were incubated in aerobic conditions in an IKA KS 4000 shaker at 120 revolutions min^−1^ at 28 °C for 24 h.

### 2.4. Ultra-High-Performance Liquid Chromatography (UHPLC) for Determination of Ethanol and Residual Glucose Concentrations

Ultra-high-performance liquid chromatography (UHPLC) with a RID detector (Agilent Technologies, Santa Clara, CA, USA) was used to determine the concentration of sugar, in this case residual glucose and ethanol as a fermentation product. The volume of the analyzed sample was 10 µL, and the flow rate of the mobile phase (0.0025 M H_2_SO_4_) was 0.6 mL min^−1^. After centrifuging the samples for 5 min at 6000 revolutions min^−1^, 750 µL of the supernatant was added to 750 µL of ZnSO_4_ × 7H_2_O solution (Gram-Mol, Zagreb, Croatia) with a concentration of 100 g L^−1^, and the resulting solution was mixed for 20 s and left at room temperature for 10 min. The samples were then centrifuged for 10 min at 16,210× *g* to precipitate proteins and impurities. The samples thus obtained were diluted with demineralized water in a ratio of 1:1, filtered through a filter with pores of 0.2 µm size, and the samples thus prepared were analyzed on a UHPLC device with a RID detector. The obtained chromatograms were processed using the computer program OpenLAB CDS, and the unknown concentrations of the detected compounds in the samples were determined according to the equations of the parallel lines (Table 1).

### 2.5. Spectrophotometric Method of Determining GSH and MDA Concentrations

After 12 and 24 h, 10 mL of the sample was removed from each Erlenmeyer flask. The extracted samples were centrifuged (7940× *g* for 10 min) to separate the yeast biomass from the supernatant. The biomass of each sample was washed twice with distilled water. Then 10% TCA (Kemika, Zagreb, Croatia) was added, and the samples were homogenized and centrifuged (7940× *g* for 10 min) to remove proteins. Then 50 µL of the supernatant was carefully separated, and 750 µL of 1 M K-phosphate buffer, pH = 7.4 and 100 µL of DTNB (Sigma Chemical Co., St. Louis, MO, USA) were added to it. Along with the samples, a sample of distilled water was also prepared, which served as a blank test. The intensity of the resulting colored GSH-DTNB complex was measured using a UV-Vis spectrophotometer at a wavelength of 412 nm (according to a blank test).

The concentration of GSH in the samples was calculated using the Lambert–Beer law from the measured absorbance and the known absorption coefficient. The molar absorption coefficient (ε) is constant for a certain compound and the wavelength at which it is measured [26]. The concentration of GSH was calculated as the ratio of the measured absorbance and the absorption coefficient multiplied by the length of the light path through the solution (Equation (1))

(1) $$c(GSH)=\frac{A}{\varepsilon \times l}$$

where:

*c*(*GSH*)—concentration of GSH in the sample;

A—measured absorbance at 412 nm;

ε—molar absorption coefficient (14.15 mM^−1^ cm^−1^);

l—the length of the path that light passes through the solution (1 cm).

In order to measure the concentration of MDA in the samples, they were prepared in the same way as for measuring the concentration of GSH until the separation of 100 µL of the supernatant, to which 800 µL of 0.6% TBA was added. In addition to the prepared samples, a blank test was also prepared that contained distilled water instead of the sample. Tubes with the reaction mixture were heated at a temperature of 90 °C for 30 min in a heating block. After heating, the tubes were cooled to stop the reaction, and the absorbance was measured using a UV-Vis spectrophotometer against a blank at a wavelength of 532 nm. The concentration of MDA in the samples was calculated according to the Lambert–Beer law and the known absorption coefficient (ε) [27] (Equation (2))

(2) $$c(MDA)=\frac{A}{\varepsilon \times l}$$

where:

*c*(*MDA*)—concentration of MDA in the sample;

A—measured absorbance at 532 nm;

ε—molar absorption coefficient (156 mM^−1^ cm^−1^);

l—the length of the path that light passes through the solution (1 cm).

### 2.6. Statistical Analysis

During the experimental part of this work, all experiments were performed in triplicate, and before statistical data processing, the results obtained during the research were prepared and edited in the Microsoft Office Excel 2016 program. One-way ANOVA followed by Tukey’s HSD post hoc test were used to analyze the impact of two different doses of OTA on the dependent variables, i.e., ethanol, residual glucose, concentrations of GSH and MDA. Statistical analysis was performed at a significance level of 0.05.

#### Artificial Neural Network (ANN) Modelling

All the ANN modelling was performed in Statistica v. 14. 0 software (StatSoft, Round Rock, TX, USA) with yeast strain, concentration of OTA and time of cultivation as inputs for ANN models, with four outputs being concentrations of GSH, MDA, ethanol and glucose. Multilayer perceptron networks with random software separation of data for training, testing, and validation in 70:15:15 ratios, respectively, with a back error propagation algorithm were tested. For ANN models that used subsampling, the sample of subsampling was set to five, with seed for subsampling set to 1000. The results were chosen in accordance with the highest R^2^ values and lowest root mean squared error (RMSE) for training, testing, and validation.

## 3. Results and Discussion

### 3.1. Effect of OTA on Ethanol Production and Residual Glucose in Medium

Although the number of scientific papers examining the effects of mycotoxins on the yeast fermentation process is extremely limited, there is evidence that mycotoxin contamination, including OTA, can affect the number of volatile compounds produced during fermentation [28]. Research has shown that yeasts have a certain sensitivity to mycotoxins, which is confirmed by the fact that mycotoxins can inhibit enzymes responsible for carrying out fermentation, delay growth [16] and cause oxidative stress [28]. The reason for the different effects of OTA on different yeast strains may be in the structure of the cell walls and the amount of mannoproteins, β-glucan and chitin and how they interact with OTA [29,30]. On the other hand, the physiological and metabolic capabilities of yeasts allow it to adapt to a wide variety of conditions, including some extreme conditions like cold, heat, dryness, acidity, alkalinity, salinity, osmolarity, UV radiation, and toxicity, or any combination of these harsh conditions [16,31,32,33]. In this work, the concentrations of ethanol and the remaining glucose were determined during cultivation for 24 h in the YPG medium without or with the addition of OTA, and the results are shown in Table 2. Table 2 shows that after 12 h of cultivation, the highest concentration of ethanol was detected in samples with yeast *S. bayanus* (4.375–4.525 mg mL^−1^), and the lowest concentration was detected in samples with *P. guilliermondii* yeast (0.430–0.620 mg mL^−1^). In all samples supplemented with OTA, except for the one with *S. bayanus*, lower ethanol concentrations were measured in comparison to the control samples. Although these differences are small, they still indicate a negative influence of OTA on ethanol production after 12 h of cultivation. Table 2 also shows the results of ethanol concentrations in samples without and with added OTA after 24 h of cultivation. As expected, ethanol concentrations after 24 h of cultivation are higher compared to ethanol concentrations after 12 h of cultivation. The highest ethanol concentrations were measured in samples with yeast *H. uvarum* (7.365–7.490 mg mL^−1^), while the lowest were measured in samples with *K. marxianus* (1.940–1.952 mg mL^−1^). Also, the largest and smallest differences in ethanol concentration after 12 and 24 h of cultivation were observed in samples with *H. uvarum* and *K. marxianus*, respectively. The obtained results point to the fact that the presence of OTA does not have a significant effect on the ability of the tested yeasts to produce ethanol, since at the end of the experiment (after 24 h), the highest concentrations of ethanol were measured in all samples, which means that the main factor for ethanol production is yeast species and not the presence, in this case, of OTA. The obtained results can be compared with the research of [34], who used a total of 20 different yeast strains of *K. apiculata* and *S. cerevisiae* and determined the percentage of ethanol produced after the first, third and seventh day of cultivation with the addition of different concentrations of OTA in the medium (0.2, 0.6, 3 and 6 µg L^−1^). Their results showed that there were no significant differences in ethanol production even at higher concentrations of OTA. In later research [35] used five genetically different strains of *S. cerevisiae* yeast that achieved a high ethanol yield (8.32–10.91 mg mL^−1^) in the presence of OTA (2 µg L^−1^). Such results indicate the low toxicity of OTA towards yeasts, i.e., the ability of yeasts to adapt to toxic conditions, and the fact that the yeasts used in the mentioned research were genetically modified, which can increase their resistance to OTA, and should also be taken into account. Even though OTA can cause oxidative stress, delay growth and inhibit fermentation, it was shown that by the end of cultivation, yeasts adapted to unfavorable conditions and could be utilized in uncontaminated conditions since even the cell count was in correlation with fermentation byproducts [16,36]. Similarly, it should be noted that in the presence of OTA, the yeast cell count is like the one of the controls after 12 h of incubation (10^7^–10^8^ cells mL^−1^), while by the end of the 24-h incubation, the final number of yeast cells exposed to OTA was 1–1.5 log more than the initial cell number, meaning that the presence of OTA had no effect on cell growth. Results obtained within this research suggest that the success of fermentation depends on the strain of yeast used, since after 24 h of cultivation with the addition of OTA, relatively large amounts of ethanol were detected in samples with two yeast species (*S. bayanus* and *H. uvarum*), while with *K. marxianus* and *P. guilliermondii* significantly lower amounts of ethanol were measured (Table 2).

In addition to determining the concentration of ethanol, the concentration of residual glucose in YPG medium was also determined during the cultivation of yeasts with and without the addition of OTA, and the results are shown in Table 3. Given that glucose was not detected in any sample with *K. marxianus* (Table 3), a much higher concentration of ethanol was expected, but this was not the case (Table 2). Such results point to the fact that during the cultivation of *K. marxianus*, different metabolic pathways take place during which glucose is consumed. It is worth mentioning that [37] investigated the metabolic characteristics of *K. marxianus* strains and showed that ethanol production is dependent on the yeast strain and carbon source, i.e., *K. marxianus* primarily ferments lactose. If we look at the concentrations of produced ethanol (Table 2) and residual glucose (Table 3) in samples with *S. bayanus*, a clear connection between these two parameters is visible. More precisely, after 12 h of cultivation, more than 9 mg mL^−1^ of glucose remained in all samples, while after 24 h of cultivation, glucose was detected only in the sample with 4 µg mL^−1^ of added OTA. Consequently, in all samples, after 12 h, less ethanol was detected compared to 24-h cultivation (Table 2). A similar trend can be observed in samples with the yeasts *P. guilliermondii* and *H. uvarum*, although the consumption of glucose and therefore the concentrations of ethanol are significantly lower compared to *S. bayanus.*

### 3.2. Effect of OTA on Oxidative Stress Parameters, GSH and MDA Concentrations

GSH is the most widespread redox molecule in eukaryotic cells, so its role in maintaining the cellular redox state is extremely important, and research has shown that GSH is an extremely important antioxidant molecule in yeasts [38]. The presence of GSH, and its reaction with harmful compounds are considered mechanisms of cellular protection [13]. Table 4 shows how the concentration of GSH differs depending on the tested yeast strain and applied OTA concentration, but also that in all control samples, between 11.84 and 12.83 µM of GSH was measured. Regarding applied OTA concentration, it is evident that lower concentrations have insignificant effect on the yeasts, since higher GSH concentrations were detected in those samples. The only exceptions were samples with *P. guilliermondii*, especially those after 24 h of incubation, where higher GSH concentrations were detected with a higher OTA concentration (4 mg mL^−1^). In the available literature, there are no data on the effect of OTA on GSH concentration in yeast cells, but similar studies are carried out on cell lines in vitro and in vivo by exposing laboratory animals to different concentrations of OTA [39,40,41]. However, [42] studied the effect of copper on two different strains of *S. cerevisiae* during the fermentation of white grape must, since high concentrations of copper cause oxidative stress by reducing the number of yeast cells and also affect enzyme activity. In their study, GSH concentrations generally increased, with some exceptions where GSH was not detected or was detected at very low concentrations. That phenomenon was explained by the fact that in the presence of copper, oxidation of GSH is accelerated and leads to the formation of a complex with glutathione disulfide (GSSH), so it was not possible to detect GSH.

Table 5 shows how the concentration of MDA changes depending on the strain of yeast used, the OTA concentration and the time of cultivation. The lowest and highest MDA concentrations were detected after 12 h of incubation in samples with *P. guilliermondii* and 2 mg mL^−1^ of OTA (0.73 µM), and after 24 h of cultivation in samples with *S. bayanus* and 4 mg mL^−1^ of OTA (1.19 µM), respectively. It can be seen that in almost all samples after 24 h of cultivation, the concentration of MDA was lower than after 12 h (Table 5). An increased concentration of MDA indicates lipid peroxidation or cell damage; therefore, higher MDA concentrations in samples with *S. bayanus*, *H. uvarum* and *P. guillermondii* indicate that those yeasts are still susceptible to the toxic influence of OTA. On the other hand, *K. marxianus* proved to be the most resistant yeast, since after 24 h of exposure to 4 µg mL^−1^ OTA, the concentration of MDA decreased significantly. As MDA is the final product of lipid peroxidation and is considered a biomarker of oxidative stress and cellular damage, the inability to detect MDA or the detection of low concentrations of MDA may be the result of low doses of the stressor (in this case, OTA) or poor sensitivity of the method used during the research [43]. Although it is a different type of research, [44] showed that an increase in GSH concentration can negatively affect the concentration of MDA in liver tissue.

### 3.3. Predictive Assessment of ANNs

In this case, for all ANN models, three parameters in terms of yeast strain, concentration of OTA and time of cultivation were used as inputs, with four outputs being the concentration of GSH, MDA, ethanol and glucose. For the purpose of testing whether ANN models could show good correlation between obtained data and their prediction, the first step was to find training, test and validation ratios that were best suited in terms of not overtraining or undertraining ANNs [45]. Different types of ratios that were tested were: 70:15:15, 60:20:20, 50:30:20 and 70:20:10. As mentioned before, for some of the ratios, undertraining or overtraining happened, meaning that, for example, in the case of the 50:30:20 ratio, ANNs showed low R^2^ values for training with high errors and high R^2^ values for testing and validation with low errors. Another case was for ratios of 70:20:10, where high R^2^ values were obtained for training with low errors, while for training and validation, low R^2^ values were obtained with high errors. The best performance was observed for ANNs that had a 70:15:15 ratio, and as in all cases, software was selected to randomly select the data for those ratios. As can be seen in Table 6, the ANN with number 1 (ANN1) was selected as the most efficient one in terms of the highest R^2^ values for training, testing and validation with the lowest errors from 100 developed ANNs. When looking at network configuration, the first number represents the number of inputs, which in our case was three, regarding yeast strain, the concentration of OTA and the time of cultivation. The last number, which is four, represents the output of ANN regarding the concentrations of GSH, MDA, ethanol and glucose. The number in the middle shows how many neurons in the hidden layer were used for this ANN. The number of neurons in the hidden layer can vary depending on how many inputs and outputs there are. In our case, in software, the range was set from 4 to 13, and as can be seen, 10 was the most efficient. Both the hidden and output activations for this ANN1 were hyperbolic tangents. In terms of R^2^ values for training of 0.9457, 0.9373 for testing and 0.9249 for validation, with root-mean-square error (RMSE) of 0.6132, 0.7263 and 1.1029, respectively, were obtained.

When looking at the results that are so promising, one has to take all aspects into consideration. The first thing is the number of experimental data points that were used for ANN, which in our case was very low (108 rows of data, making the data matrix 7 × 108). Usually, for very precise ANNs there are more than thousands of experiments in order to achieve more stable ANNs with more precise predictions [46]. The reason for the lower number of experiments lies in their financial cost. Before doing more experiments, we wanted to test whether it was possible to use this experimental data to get good predictions with ANNs. Now that the results are promising, we will continue with new experiments that will expand this database. Another aspect that has to be taken into consideration is that in recent times, when people are using more and more ANNs, questions have arisen about their validity [47]. That is the reason why some sort of cross-validation is necessary to confirm whether the ANNs are overtrained or undertrained. For that purpose, we performed subsampling with the data subsampling set to five and with the same ratio for subsampling as for ANN1 of 70:15:15 for the training, test and validation, respectively. The number of neurons in the hidden layer was also fixed to 10, as for ANN1, with the seed for subsampling set to 1000. The results for five ANNs that were obtained in terms of R^2^ values ranged from 0.7697 to 0.9536 for training, 0.7348 to 0.9273 for testing and 0.7585 to 0.9133 for validation. The ANN with the highest R^2^ values and lowest errors obtained with subsampling is presented in Table 6, number 2 (ANN2). When looking at the results for ANN1 and ANN2, it is visible that even with subsampling, the results are promising, with a difference of only 1% less than from the ANN without any cross-validation.

**Figure 1:**
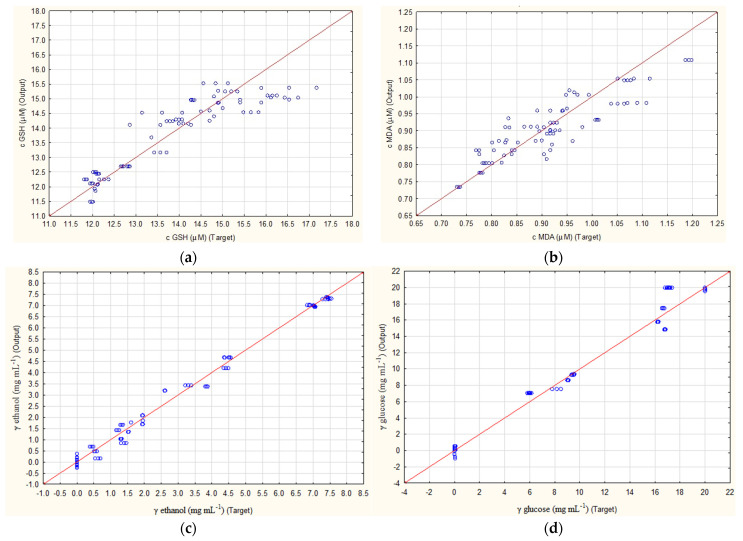

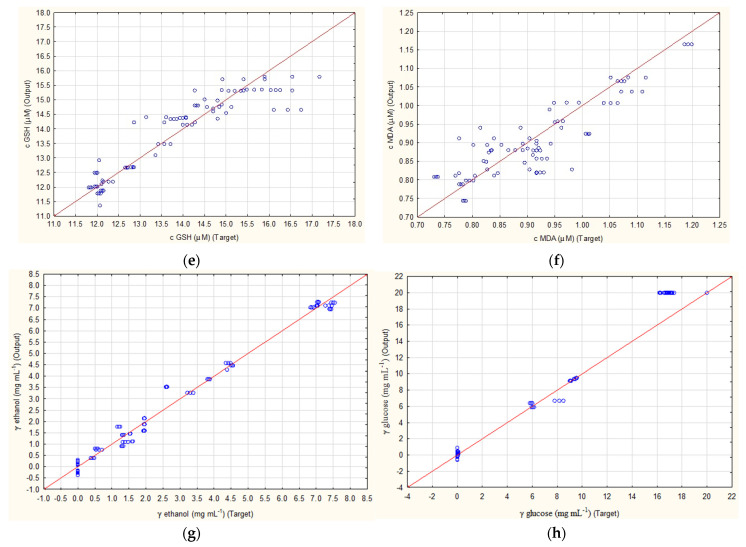
Prediction of ANN1 (without subsampling) for concentration of (**a**) GSH, (**b**) MDA, (**c**) ethanol, (**d**) glucose and ANN2 (with subsampling) for concentration of (**e**) GSH, (**f**) MDA, (**g**) ethanol and (**h**) glucose.

The results of how well ANN1 and ANN2 were able to predict the concentrations of four outputs (concentration of GSH, MDA, ethanol and glucose) are presented in Figure 1. For both ANNs, the highest R^2^ values were obtained for ethanol prediction. While ANN1 had values of 0.9955, 0.9822 and 0.9973 (Figure 1c), ANN2 had values of 0.9945, 0.9945 and 0.9884 (Figure 1g) for training, testing and validation. Furthermore, for the prediction of glucose concentrations, values of 0.9949, 0.9872 and 0.9958 were achieved for ANN1 (Figure 1d) and values of 0.9921, 0.9923 and 0.9872 for ANN2 (Figure 1h). Both cases show that, with such high R^2^ values, these ANNs could be used for quantification purposes. The lowest results were obtained for the prediction of MDA concentrations, which for ANN1 were 0.8679, 0.8394 and 0.8399 (Figure 1b) and for ANN2 were 0.9078, 0.7493 and 0.7845 (Figure 1f) for training, testing and validation. Somewhat better results were obtained for GSH concentration, where ANN1 achieved R^2^ values of 0.9245, 0.9403 and 0.8668 (Figure 1a) and ANN2 0.9201, 0.9730 and 0.8930 (Figure 1e).

### 3.4. Predictive Assessment of GSH, MDA, Ethanol and Glucose Concentrations Based on Hypothetical OTA Concentrations and Hours of Incubation

In order to test the predictions for the same four yeast strains with different concentrations of OTA and times of cultivation from experimental ones, for both ANN1 and ANN2, predictions based on their original data were performed. In terms of experiment duration, instead of 12 and 24 h, 6, 18 and 30 h were selected and for concentrations of OTA, instead of 0, 2 and 4 µg mL^−1^ concentrations of 1, 3 and 10 µg mL^−1^ were selected. Results for the prediction of newly selected experimental parameters are presented in Table 7.

Table 7 presents the results that were obtained for prediction based on newly selected parameters for ANN1 and ANN2, in which, when some of concentrations had negative values, for example ethanol and glucose concentration during cultivation of selected yeasts, they were marked with *, since negative concentrations are not realistic.

The maximum ethanol concentration was predicted for *H. uvarum* after 30 h of cultivation in the presence of 1 and 3 µg mL^−1^ of OTA with ANN2 model and amounted 7.53 mg mL^−1^. Within that prediction model, ethanol concentration usually increased with time of cultivation, no matter the OTA concentration in the samples, with exceptions being samples with *S. bayanus* and *K. marxianus*. The same can be seen for the ANN1 model, with the exceptions being samples with *K. marxianus* and *P. guilliermondii*. However, these predictions differ from the results obtained by measurements within this work since in all samples ethanol concentration increased with time no matter the OTA concentration, even though in some samples this was not significant (*K. marxianus*). This also proves, as stated before, that for good ANN predictions more experiments are needed in order to form a qualitative database which can then be used for such predictions. It is also worth pointing out that the ANN2 model predicted very low ethanol concentrations in samples with 10 mg mL^−1^ of OTA, while in the ANN1 model, the OTA concentration did not affect ethanol production.

During the experiment, as ethanol concentration in samples increased (Table 2), glucose concentration decreased (except in samples with *K. marxianus*, where it was not even detected) (Table 3). The same can be seen in Table 7, as both models predicted the same general trend.

The ANN2 model also predicted a decrease in GSH concentration for *S. bayanus* and *H. uvarum*, which is consistent with results obtained in this experiment during cultivation with 2 µg mL^−1^ of OTA. However, at higher OTA concentrations, higher GSH concentrations are predicted, which indicates that no oxidative stress should occur. On the other hand, ANN1 predicts that at lower OTA concentrations, GSH concentration increases (the only exceptions are *S. bayanus* and *K marxianus* at 1 µg mL^−1^ and 3 µg mL^−1^ of OTA, respectively), while at 10 µg mL^−1^ of OTA, GSH concentration increases, with the only exception being *K. marxianus*.

MDA concentrations are predicted to increase at lower levels of OTA (with the exception being *K. marxianus*) according to the ANN1 model, and the same trend is happening during this experiment (Table 5). The same general trend can be seen for the obtained results as well as the predictions of ANN1. In contrast, the ANN2 model predicts that at lower concentrations of OTA, MDA concentration is decreasing, with the exception being *H. uvarum* (Table 7). The most interesting prediction is the decrease of MDA in all samples with high OTA concentration (10 µg mL^−1^) because that can refer to no oxidative stress in cells.

## 4. Conclusions

A wide variety of harmful conditions such as cold, heat, acidity, dryness, salinity and presence of toxic metabolites have negative effects on different groups of microorganisms. However, yeasts are one of the most durable groups and their physiological and metabolic capabilities allow them to adapt to one or even more different harmful conditions. Even though they showed some sensitivity to mycotoxins, in the presence of OTA, yeasts also adapted to these conditions, since OTA had no significant effect on the concentration of ethanol or GSH and MDA concentration. The results of this study show that OTA does not show effects on ethanol production, which suggests the tolerance of yeasts to this type of toxic stressor. However, OTA at a higher concentration (4 µg mL^−1^) induced the production of oxidative stress parameters. During cultivation, some differences were detected, and those differences were mainly related to the yeast strain used. However, since there are very little available data on the effects of OTA on oxidative stress parameters and fermentation ability of yeasts, future experiments are needed and planned. In order to minimize the costs of the experiments, ANNs were used to predict in which direction future experiments can and will go on.

## Figures and Tables

**Table 1:** Equations of basic directions for determining the concentration of compounds by ultra-high performance liquid chromatography (UHPLC).

Compound	Retention Time, *t*_R_ (min)	The Equation of the Perpendicular Direction	R^2^
Ethanol	11.072	y = 55,421x + 1343	1.0000
Glucose	4.821	y = 135,278x − 3377	0.9997

**Table 2:** Ethanol concentrations (mg mL^−1^) after 12 and 24 h of selected wine yeast strains in the presence of OTA.

Sample	γ OTA (µg mL^−1^)	γ Ethanol (mg mL^−1^)	
		12 h	24 h
*S. bayanus*	0	4.415 ± 0.092 a	7.050 ± 0.028 a
	2	4.525 ± 0.035 a	7.020 ± 0.028 a
	4	4.375 ± 0.007 a	6.870 ± 0.057 a *
*K. marxianus*	0	1.603 ± 0.007 b	1.952 ± 0.007 b
	2	1.529 ± 0.009 b	1.948 ± 0.008 b
	4	1.320 ± 0.030 b	1.940 ± 0.010 b
*H. uvarum*	0	1.390 ± 0.113 b	7.365 ± 0.134 a
	2	1.305 ± 0.035 b	7.490 ± 0.071 a
	4	1.205 ± 0.064 b	7.410 ± 0.042 a
*P. guilliermondii*	0	0.620 ± 0.113 c	2.605 ± 0.021 c *
	2	0.430 ± 0.071 c	3.845 ± 0.049 c
	4	0.545 ± 0.064 c	3.295 ± 0.120 c

* Significant difference within group. Values with different letters in the same column are significantly different (*p* < 0.05).

**Table 3:** Concentrations of residual glucose in the medium (mg mL^−1^) after 12 and 24 h of cultivation of selected wine yeast strains in the presence of OTA.

Sample	γ OTA (µg mL^−1^)	γ Glucose (mg mL^−1^)	
		12 h	24 h
*S. bayanus*	0	9.545 ± 0.021 a	n.d. a
	2	9.035 ± 0.078 a *	n.d. a
	4	9.400 ± 0.085 a	0.060 ± 0.057 a
*K. marxianus*	0	n.d. b	n.d. a
	2	n.d. b	n.d. a
	4	n.d. b	n.d. a
*H. uvrum*	0	16.67 ± 0.127 c	0.045 ± 0.021 a
	2	16.25 ± 0.064 c *	0.050 ± 0.014 a
	4	16.79 ± 0.071 c	0.065 ± 0.007 a
*P. guilliermondii*	0	17.20 ± 0.198 c	8.145 ± 0.502 c *
	2	16.95 ± 0.240 c *	5.890 ± 0.127 c
	4	17.16 ± 0.283 c	6.050 ± 0.113 c

n.d.—not detected. * Significant difference within group. Values with different letters in the same column are significantly different (*p* < 0.05).

**Table 4:** GSH concentrations after 12 and 24 h of cultivation in the presence of two concentrations of OTA.

Sample	γ OTA (µg mL^−1^)	GSH Concentration (µM)	
		12 h	24 h
*S. bayanus*	0	11.98 ± 0.04 a	12.26 ± 0.11 a *
	2	16.15 ± 0.11 a *	14.84 ± 0.28 a
	4	13.36 ± 0.00 a	15.41 ± 1.30 a
*K. marxianus*	0	11.84 ± 0.04 a *	11.98 ± 0.04 a *
	2	15.19 ± 0.14 a	13.60 ± 0.46 a
	4	14.10 ± 0.11 a	13.57 ± 0.14 a
*H. uvarum*	0	12.05 ± 0.04 a *	12.69 ± 0.04 a
	2	16.43 ± 0.32 a	16.54 ± 0.64 a *
	4	14.31 ± 0.04 a	13.78 ± 0.07 a
*P. guilliermondii*	0	12.12 ± 0.04 a *	12.83 ± 0.04 a *
	2	13.57 ± 0.71 a	15.65 ± 0.18 a
	4	13.99 ± 0.07 a	15.41 ± 0.05 a

* Significant difference within group. Values with different letters in the same column are significantly different (*p* < 0.05).

**Table 5:** MDA concentrations after 12 and 24 h of cultivation in the presence of two concentrations of OTA.

Sample	γ OTA (µg mL^−1^)	MDA Concentration (µM)	
		12 h	24 h
*S. bayanus*	0	0.92 ± 0.01 a	0.95 ± 0.07 a
	2	0.83 ± 0.03 a	0.92 ± 0.03 a
	4	0.88 ± 0.01 a	1.19 ± 0.01 a
*K. marxianus*	0	0.92 ± 0.00 a	0.78 ± 0.01 a
	2	0.97 ± 0.02 a	0.79 ± 0.01 a
	4	1.05 ± 0.01 a	0.89 ± 0.07 a
*H. uvarum*	0	0.93 ± 0.01 a	0.79 ± 0.01 a
	2	0.83 ± 0.00 a	1.09 ± 0.02 a
	4	1.07 ± 0.01 a	0.80 ± 0.04 a
*P. guilliermondii*	0	0.92 ± 0.01 a	0.90 ± 0.08 a
	2	0.73 ± 0.01 a	0.84 ± 0.06 a
	4	1.01 ± 0.01 a	1.08 ± 0.03 a

Values with different letters in the same column are significantly different (*p* < 0.05).

**Table 6:** Characteristics of ANN for prediction of GSH, MDA, ethanol and glucose concentrations.

Number	Network Configuration	Training	Training Error	Test	Test Error	Validation	Validation Error	Hidden Activation	Output Activation
1	3-10-4	0.9457	0.6132	0.9373	0.7263	0.9249	1.1029	Tanh	Tanh
2	3-10-4	0.9536	0.8801	0.9273	0.9729	0.9133	1.9684	Tanh	Tanh

**Table 7:** Predictive assessment of GSH, MDA, ethanol and glucose concentrations based on hypothetical OTA concentrations and hours of incubation.

Yeast Strain	t (h)	c OTA (ug/mL)	ANN1				ANN2			
			c GSH (µM)	c MDA (µM)	(mg/mL)	c Glucose (mg/mL)	c GSH (µM)	c MDA (µM)	c Ethanol (mg/mL)	c Glukose (mg/mL)
*S. bayanus*	6	1.00	15.59	0.85	0 *	11.28	15.50	0.85	0 *	10.66
*S. bayanus*	6	3.00	14.40	0.85	0.06	11.87	15.05	0.94	0 *	9.96
*S. bayanus*	6	10.00	10.00	1.09	1.88	10.83	16.17	1.14	0 *	0 *
*S. bayanus*	18	1.00	15.56	0.80	6.57	4.24	15.91	0.84	7.34	5.08
*S. bayanus*	18	3.00	14.90	0.99	6.63	4.56	14.39	0.94	7.35	5.94
*S. bayanus*	18	10.00	9.53	1.09	6.10	7.13	17.11	0.26	0.00	0 *
*S. bayanus*	30	1.00	15.96	0.88	7.05	0 *	14.07	0.80	0.18	0 *
*S. bayanus*	30	3.00	15.62	1.09	7.07	0 *	14.98	1.06	0 *	0 *
*S. bayanus*	30	10.00	13.06	1.19	7.07	0 *	17.09	0.26	0 *	0 *
*K. marxianus*	6	1.00	15.39	0.92	0 *	17.42	14.69	0.85	0 *	20.00
*K. marxianus*	6	3.00	15.12	1.05	0 *	16.46	14.86	1.01	0.35	20.00
*K. marxianus*	6	10.00	13.37	1.17	1.22	10.39	15.44	0.96	0.00	19.99
*K. marxianus*	18	1.00	15.44	0.94	2.63	0 *	15.49	0.91	4.17	0 *
*K. marxianus*	18	3.00	14.24	0.89	2.59	0.01	14.30	0.94	4.78	0 *
*K. marxianus*	18	10.00	9.30	0.94	1.32	0 *	17.03	0.26	0 *	0 *
*K. marxianus*	30	1.00	14.62	0.62	1.04	0 *	14.28	0.54	0 *	0 *
*K. marxianus*	30	3.00	13.82	0.85	0.72	0 *	13.85	0.66	0 *	0 *
*K. marxianus*	30	10.00	9.23	1.10	0 *	0 *	17.06	0.26	0 *	0 *
*H. uvarum*	6	1.00	14.62	0.67	1.03	20.00	14.23	0.80	0.15	20.00
*H. uvarum*	6	3.00	14.64	0.88	1.00	20.00	14.41	0.89	0.61	20.00
*H. uvarum*	6	10.00	13.70	1.02	2.16	20.00	14.98	0.33	0.00	20.00
*H. uvarum*	18	1.00	15.08	0.78	5.57	2.55	15.25	0.86	2.83	3.10
*H. uvarum*	18	3.00	15.07	1.00	5.65	2.95	15.42	1.08	4.08	2.71
*H. uvarum*	18	10.00	12.96	1.02	6.99	2.48	16.98	0.26	0 *	0 *
*H. uvarum*	30	1.00	15.33	0.80	7.45	0 *	16.20	0.91	7.53	2.88
*H. uvarum*	30	3.00	14.43	0.75	7.46	0 *	15.19	0.56	7.53	3.48
*H. uvarum*	30	10.00	9.05	0.42	7.46	2.86	17.10	0.26	0 *	0 *
*P. guilliermondii*	6	1.00	14.43	0.82	0.41	20.00	14.26	0.81	0 *	20.00
*P. guilliermondii*	6	3.00	14.22	0.85	0.61	20.00	14.46	0.86	0 *	20.00
*P. guilliermondii*	6	10.00	13.43	0.92	1.11	20.00	15.02	0.27	0 *	20.00
*P. guilliermondii*	18	1.00	14.59	0.83	0 *	16.17	14.46	0.78	1.16	20.00
*P. guilliermondii*	18	3.00	14.70	1.00	0 *	14.63	14.61	0.92	1.88	20.00
*P. guilliermondii*	18	10.00	14.33	1.11	1.11	12.71	16.83	0.26	0.00	20.00
*P. guilliermondii*	30	1.00	15.05	0.83	6.98	2.73	15.73	0.76	7.20	0.45
*P. guilliermondii*	30	3.00	15.19	1.05	6.98	3.33	16.16	0.97	7.09	0 *
*P. guilliermondii*	30	10.00	12.70	0.86	7.40	4.48	17.06	0.26	0 *	0 *

* predicted values were negative, therefore they were replaced with 0.

## Data Availability

The data are contained within the article.
